# An Experimental Study of the Influence of Diet on Teeth Formation*Reprinted from “British Dental Record.”

**Published:** 1919-05

**Authors:** May Mellanby


### AN EXPERIMENTAL STUDY OF THE INFLUENCE OF DIET ON TEETH FORMATION *

BY MAY MELLANBY

The misery caused, both directly and indirectly, by unsound teeth, is too well known to the medical profession and to the general public to need emphasis. The fact that dentistry plays such an important part in civilized communities today proves that the problem needs the further attention of medical research.

The majority of investigations so far carried out have dealt with the etiology of caries. The type of food, considered from the point of view of its hardness and softness, its liability to undergo bacterial decomposition, with the production of acids, toxins, etc., has completely dominated the picture.

### VIEWS ON ETIOLOGY OF DENTAL HYPOPLASIA

The etiology of hypoplasia has received less attention. Several suggestions have, however, been put forward to account for the imperfect development of teeth. A particular defect of certain teeth has been associated with congenital syphilis; the exact method by which it is produced has not been demonstrated. Other types of hypoplasia, characterised by one or more bands of defective enamel, have been traced to one of the specific fevers or to some other acute illness. As regards the more generalized types of gross hypoplasia, the parts of the teeth usually affected point to some cause acting during the first two years or so of life. The association with infant feeding is obvious, but here again it is not known whether the results are due to insufficient food, diet of an unsuitable kind, the assimilation of harmful products, or intestinal

* Reprinted from "British Dental Record."

disturbances set up by abnormal fermentative processes reacting on the trophic functions which control calcification.

Apart from these gross forms of hypoplasia, there is little doubt that marked differences exist in the degree of hardness of the enamel, the physical structure, and the perfection of calcification. The so-called "mottled teeth" exhibit in their partial lack of translucency such slight forms of hypoplasia. Between this and perfect enamel there must be many grades. Another hypothesis that also needs further investigation is, that defective calcification is due to a deficiency of calcium salts in the diet.

### SCOPE OF PRESENT INVESTIGATION

The investigation to be described in this paper was undertaken primarily with the object of studying—

1. The factors involved in the development of sound teeth and of the growth of the jaws in relation to the size of the teeth.

2. The factors bearing on the immunity of erupted teeth to caries and other diseases.

In this preliminary paper I propose to describe experimental work which deals with the first of the above problems, and will adduce evidence to show that:

1. Hypoplasia of the teeth is caused largely by a deficient diet.

2. The factor in the diet which controls the calcification of the teeth is something of the nature of an accessory food factor (vitamine).

3. This factor has a similar distribution to that of fat-soluble A, and has recently been shown by E. Mellanby to be largely responsible for the calcification of bone, a deficiency of this substance in the diet being followed by the development of rickets.

Experiments, dealing with conditions affecting the teeth after eruption, are in progress, but it will be agreed that this question is in many ways of secondary importance; for if the enamel on all parts of the crowns of teeth is abundant and sound, and if the teeth are adequately spaced, then such teeth are less likely to be attacked by caries and other diseases.

The part of this work to be described here has involved the examination of the teeth of those puppies used by my husband, Dr. Edward Mellanby,* in the course of an extensive research on rickets made for the Medical Research Committee. Details of the diets will be found in a paper to be published immediately by him. Suffice it here to say that the diets consisted chiefly of white bread and separated milk, about 200 c.m. per diem, together with the substances to be tested.

For work of this nature domesticated dogs are eminently suitable, in the first place because their normal diet is very similar to that of man, and in the second place because the formation and development of their teeth is more comparable with his than is the case with most other available animals.

In examining the jaws and teeth attention has been paid to the study of (1) the time of shedding of the deciduous teeth; (2) the time of eruption of the permanent dentition; (3) the arrangement of the teeth in the jaws; (4) the condition of the enamel, etc.; (5) the calcium content of the teeth.

### ARRANGEMENT OF TEETH IN JAWS

(a) and (b) Normal.

(c) Lower incisors irregular and somewhat crowded together.

(d), (e), and (f) Upper incisors fairly regular, but lower ones—(f) very irregular and crowded.

### CONDITION OF ENAMEL OF PERMANENT TEETH

(a) White and shiny.

(b) On whole white and shiny; back teeth very slightly yellow.

(c) Dark brown coloring on that part of incisors near neck, especially of upper incisors; enamel on greater part of carnassials and first molars dull brown.

(d) The enamel does not appear very good on any of the teeth, but it is especially defective on first incisors and first molars of upper jaw and on that part of lower carn, near neck.

(e) The enamel fairly good on all permanent teeth.

(f) The enamel on all the teeth is very defective, except perhaps on tips of some of the cusps.

These experiments and others of the same type show that when linseed oil is the only fat in the diet, then there is a delay in the shedding of the deciduous teeth and in the development of the permanent dentition. The animal fats, and more particularly cod-liver oil, cause these changes to proceed in a normal way. Again, the arrangement of the teeth and their appearance is much better when animal fats have been eaten.

### CONSIDERATION OF POSSIBLE INFLUENCE OF ILL-HEALTH

A criticism to be met in this type of work is that any condition of ill-health during the period of the experiment may have been responsible for the changes described. It is true that these puppies suffered from mild attacks of distemper and that none of them grew very rapidly (on the average 2 kg. in the 15 weeks). Their periods of mild illness and their rates of growth were, however, practically speaking, identical and could, therefore, not be the cause of the variations observed in the dentition.

In order to meet such a criticism in a more thorough way I will consider another set of experiments. See Table II. Here again the lower jaws of three puppies—(d), (e), and (f)—of the same litter are shown. They are a larger breed of dog than those shown in Table I. The puppies were in this case also about 8 weeks old when the experiment, which lasted 15 weeks, was started. The source of fat in each case was 10 g. of linseed oil per diem. Their relative rates of growth in the 15 weeks can be seen in the following table:

TABLE I

| | Weight at beginning. | Weight at end. | Increase. |
|---|---|---|---|
| (d) | 2490 g. | 5820 g. | **3330 g.** |
| (e) | 2675 g. | 6820 g. | 4145 g. |
| (f) | 3690 g. | 8825 g. | 5135 g. |

It must be stated here that although (e) was on the same articles of diet as (d) and (f) for 6½ weeks, yet, from this time until the end of the experiment, 10 g. of butter were given daily in addition to the linseed oil.

It will be seen that all the teeth in Table II are delayed, not so much in the loss of the deciduous teeth as in the complete eruption of the secondary dentition. More striking still is the amount of defective enamel. In (f) there is hardly any white enamel, and that present is so soft that it can be cut with a scalpel. There is considerable irregularity in the setting of the teeth, especially in (f). In fact, the teeth of (f), the most rapidly growing puppy, are in every way worse than those of (d). In (e) the bad effects of the diet have been saved to some extent by the addition of butter after the first few weeks. Then, again, the teeth of (d) are distinctly worse than those of (c), which grew comparatively slowly.

As a general rule, it is difficult to associate rapid growth with ill-health, and yet we have seen in these experiments that the teeth of the more rapidly growing puppies are worse, whether the comparison is confined to the same or to another family. It seems impossible, therefore, to escape the conclusion that the malformed teeth are the result of some specific influence of the diet, such as has been suggested.

### CALCIUM CONTENT OF TEETH

If we consider next one aspect of the chemical side of the problem, we see (Table II) that the calcium content of the teeth may be influenced by the diet, although the actual intake of calcium salts would appear to be adequate.

TABLE II

| No. of exp. | Special factor in diet. | Tooth taken. | Wt. of dry tooth. | Total CaO. | % CaO. |
|---|---|---|---|---|---|
| 90 | Suet | Lower carn. | 1.451 g. | 0.507 g. | 36.4 |
| 90 | Suet | 3 l. incisors | 0.438 g. | 0.156 g. | 36.1 |
| 94 | Yeast | Lower carn. | 0.851 g. | 0.296 g. | 34.8 |
| 101 | Butter | Lower carn. | 1.291 g. | 0.440 g. | 34.0 |
| 122 | Calcium phosp. | Lower carn. | 0.601 g. | 0.186 g. | 30.9 |
| 122 | | 3 l. incisors | 0.188 g. | 0.059 g. | 31.8 |
| 143 | Linseed oil | Lower carn. | 0.851 g. | 0.260 g. | 30.5 |

It will be seen from the above table that there is a great variation both in the weight of the teeth and in the calcium content (reckoned as calcium oxide). For example, when either suet or butter is added to the diet the carnassials weigh 1·451 g. and 1·291 g. respectively, the calcium content being 0·507 g. and 0·44 g. CaO, while when the fat is linseed oil the weight of the tooth is 0·851 g. and the calcium content reckoned as CaO is 0·260 g.

CAUSATION OF LOW CALCIUM CONTENT OF TEETH

It may be suggested that the low calcium content in this and in other similar experiments is due to a deficiency of calcium in the diet. We should certainly expect that the calcification of the teeth and bones would be imperfect if the calcium content of the diet of young animals were deficient or even on the border-line of the animals' needs. This explanation, however, does not seem to cover the experiments here described. For instance, comparing Experiments 90, 101, and 143 (Table II), the calcium content of the diet was, practically speaking, identical, and yet the calcification of the teeth is good in the first two cases and poor in the last. Then, again, referring to the puppies (a)-(f), the amount of calcium given was the same in each case, and yet the condition of the teeth was very different, and I have no doubt but that the calcium content of the teeth will prove to be greater in (a) and (b) than in any of the others, and that it will be least in (f).

The only explanation on the calcium deficiency hypothesis is that the calcium salts are not equally absorbed into the blood stream, owing to the action of the different fats. The following experiments would seem to negative this criticism.

In Experiment 122 (Fig. 4 (k) and Table II) 5 g. of calcium phosphate were added daily to the diet, the fat of which was linseed oil. The experiment lasted about 12 weeks. The deciduous teeth were late in being shed and the permanent ones were late in erupting. The enamel on many of the teeth was either defective or absent. The calcium content of one lower carnassial was found to be only 0·186 g. (CaO), the weight of the tooth being 0·601 g.

There is always the possibility that the puppy was unable to make use of the calcium in this form. To exclude this, experiments are now in progress in which the quantity of separated milk in the diet has been raised to 400 c.cm. and more daily. The teeth, in the case of one of these puppies which has been dieted for nearly three months, are poorly formed, and there is, so far, no evidence that the increased calcium in the diet, although given in a natural form, has improved matters.

There does not seem much doubt, then, that the puppies received an adequate supply of calcium in a form which ensured access to the blood. There is evidence, however, that the ameloblasts and other cells, which normally regulate the deposition of calcium in the developing teeth, are unable to perform this function successfully, owing to the lack of one or more factors in the blood stream which normally regulate this function. This factor can not be either proteins, carbohydrates, fats, or salts, but is possibly something of the nature of an accessory food factor. Whether this accessory factor acts directly on the special cells or indirectly through some other tissues, such as the ductless glands, remains to be seen.

### EFFECT OF TIME AT WHICH DIET IS STARTED

The appearance of the permanent teeth resulting from these deficient diets suggests that the extent to which the enamel, etc., is defective depends on the time at which the diet was started. In the human being the individual teeth calcify at different periods, starting before birth and ending at about 18 years of age. In puppies these changes cover a much shorter period of life; consequently one would not expect the picking out of specific teeth to be so prominent in puppies as it is in children. Nevertheless, even in puppies it is seen that in some teeth the enamel appears normal, while on others it is defective.

In the experiments so far completed the teeth most commonly affected are the lower carnassials. An examination of some of these teeth is very interesting. The enamel on the apex of the cusps may be white and shiny, while that on the rest of the tooth is either colored or absent. The carnassial looks like a snow-capped mountain, for the only trace of white enamel is on the tip of the large cusps.

There is some evidence to show that, if the diet is only started when the puppy is over 3 months old, then the development of the teeth is little, if at all, affected. For instance, in Experiment 104, the puppy was about 14 weeks old when the diet, containing linseed oil as the fat, was started. The teeth are white and shiny, and they are regularly arranged in the jaw. One lower carnassial weighed 1·412 g. and the CaO content was 0·501 g.

### THE QUESTION OF AN ACCESSORY FOOD FACTOR

It is always possible that chewing hard crusts, bones, etc., may improve the circulation in the developing jaws and so cause better teeth formation, but it can not explain the results described in this paper, for in no case was hard food of any kind given. From the point of view of bacterial action and the production of toxic products, the diets would appear, in most cases, to be similar, except in so far as the fats are concerned.

What, then, is the factor (or factors) in the diet responsible for these changes? We have seen that the puppies fed on animal fats, such as cod-liver oil, butter, and suet, had the best teeth, whereas those fed on linseed oil had the worst. There is evidence that other vegetable oils —for instance, arachis oil and cotton-seed oil—also have deleterious effects.

As has been already suggested, the metabolic factors normally involved in the process of teeth development may be of the nature of accessory food factors. It is now recognized that fat-soluble A is present in abundance in cod-liver oil, butter, and animal fats generally, and is deficient in vegetable fats. The whole subject of accessory food factors, and the part they play in health and disease, is in its infancy, and therefore can not be accepted as explaining in an unquestionable way facts such as are here described. But for the want of a better mode of expressing the general results of these experiments, one may say that a deficiency of fat-soluble A in the diet is accompanied by abnormal development of the teeth.

### DEFECTIVE TEETH AND RICKETS IN CHILDREN

A child's diet is most likely to be deficient in fat-soluble A from the ninth month to about the second year of its life; that is, at the transition period between a complete milk diet and one approximating to that of the adult. The permanent teeth calcifying at this period are the incisors, the canines, and the first molars, and it is just these teeth in children that are most commonly hypoplastic and carious. Since rickets is commonest in children during these early years, it has been suggested by Dick and others that this disease is the cause of these special teeth being so commonly defective. The work described in this paper, taken in conjunction with the experiments of E. Mellanby on rickets, puts on to an experimental basis the intimate connexion between this disease and hypoplasia of the teeth.

In some cases even the deciduous teeth of children are hypoplastic and carious. Now, the crowns of these teeth are developed, for the most part, before birth and in the first few months of extra-uterine life, and therefore the child must depend on the mother for any necessary food factors. McCollum has shown that the animal body has not the power of synthesising these factors, although it has a limited power of storage. In order to supply her offspring after the first part of her pregnancy the mother must have an adequate supply of all the necessary accessory factors in her diet. If the accessory food factor theory is the real explanation of the facts described in this paper it ought to be possible to produce similar changes in the deciduous teeth of puppies by feeding a pregnant bitch on those diets which have been shown to prevent normal tooth development in young animals. Experiments on these lines are now being carried out.

### HARMFUL NATURE OF MODERN DIETARY IN REGARD TO THE TEETH

One can now see a rather obscure but still a real picture illustrating, in part at any rate, the cause of the defective teeth of modern civilization. A perusal of a list of the substances containing fat-soluble A makes it clear that civilized conditions, and more particularly those conditions met with in urban life, exaggerate the part played in the dietary by just those substances which are deficient in this type of accessory factor. Our diet, particularly that of the poor, is now more than ever made up of specially prepared cereals, such as wheat, rice, oats, etc. Meat and the animal fats tend to play a smaller part. Vegetable fats, in the form of margarines, etc., are superseding butter, whole milk is being more and more excluded from the diet of the poor, partly at present because of the cost and partly because of a curious antipathy that many people have towards it. Then, again, with the development of civilization, breast-feeding is not so commonly practiced, and, when practiced, is often continued for only a short time. On the accessory food factor theory one can also understand why the Esquimaux in his own country, where flesh and blubber are the staple articles of diet, has such excellent teeth, while the inhabitants of Chili, who live chiefly on cereals, have teeth which are much less sound.

There is no doubt that our modern dietary is harmful as far as the teeth are concerned, and, if the results of the present work and the deductions made are correct, the teeth of the people of this country will tend to become worse, unless our diet consists in the future more of whole milk and other foods containing fat-soluble A, and less of bread, rice, potatoes, etc., which are deficient in this factor.

### SUMMARY OF RESULTS

1. A diet containing in abundance those articles with which the fat-soluble A accessory food factor is associated —e.g., cod-liver oil, butter, etc.—allows the development in puppies of sound teeth.

2. A diet otherwise adequate but deficient in the substances with which fat-soluble A is associated, brings about the following defects in puppies' teeth: (a) Delayed loss of deciduous teeth. (b) Delayed eruption of the permanent dentition; in some cases the delay in the eruption of the permanent teeth is more marked than the delay in the loss of the deciduous teeth. (c) Irregularity in position and overlapping, especially of the incisors. (d) Partial absence of or very defective enamel. (e) Low calcium content; the deficiency in calcium salts may result in the teeth being so soft that they can be cut with a scalpel.

3. The evidence makes it clear that this is an instance of diet affecting the teeth from the inside and is independent of bacterial sepsis and other oral conditions associated with food.

4. These results can not be considered as being due to acute illness or "malnutrition," for (a) the improvement to the teeth by the addition of fat-soluble A containing substances (animal fats, etc.) is as characteristic as the deleterious effect of a deficient diet; (b) there is evidence that the defective teeth are most pronounced in the rapidly growing puppies, and it is difficult to associate rapid growth with illness or "malnutrition," as generally understood.

5. This work, taken in conjunction with the experiments of E. Mellanby on rickets puts the close relationship between hypoplastic teeth and rickets on to an experimental basis.

